# Fever Hospitals in Large Towns

**Published:** 1887-08-06

**Authors:** 


### FEVER HOSPITALS AND LARGE TOWNS.

The people of Salford are very angry at the proposal on the part of the Corporation to sell a plot of ground for the purpose of establishing a fever hospital on vacant land at Hope. There is a great deal of foolish and unnecessary fear on the part of the public with regard to the danger arising from fever hospitals. Houses built within fifty yards of such institutions are probably as little likely to take harm from them as if they were built fifty miles away. The good people of Salford doubtless never expect to have fever themselves. If they did but know, they are exposed to much less risk by the gathering together of fever cases in a fever hospital than if those same cases were distributed all over the town. Truly a little knowledge is a very dangerous thing, and these public scares are very ridiculous sights.

